# Alkyl Substituent in Heterocyclic Substrate, Carbon Skeleton Length of *O*-Nucleophilic Agent and Conditions Influence the Product Composition from Competitive Reactions of *S_N_^ipso^* Substitution by Aliphatic Oligoethers

**DOI:** 10.3390/ma16227068

**Published:** 2023-11-07

**Authors:** Konstantin K. Bosov, Ekaterina V. Pivovarova, Irina A. Krupnova, Gennady T. Sukhanov, Anna G. Sukhanova, Yulia V. Filippova

**Affiliations:** Laboratory for Chemistry and Technology of High-Energy Azoles, Institute for Problems of Chemical and Energetic Technologies, Siberian Branch of the Russian Academy of Sciences (IPCET SB RAS), Biysk 659322, Russia; pivovarova.ekaterina@inbox.ru (E.V.P.); irinka-krupnova@mail.ru (I.A.K.); suhanovlab7@mail.ru (G.T.S.); nika7_anna@mail.ru (A.G.S.); filippova-yulia@mail.ru (Y.V.F.)

**Keywords:** 1-alkyl-5-nitro-1,2,4-triazole, aliphatic oligoethers, terminal OH groups, polyethylene glycol, *S_N_^ipso^*-substitution of nitro group

## Abstract

Using ^1^H NMR spectroscopy, we studied the relative mobility of the NO_2_ group in 1-alkyl-5-nitro-1,2,4-triazoles in the reaction of nucleophilic heterocyclic substitution by aliphatic oligoethers. The main pathways of the *S_N_^ipso^* substitution process and the composition of resultant products from competitive reactions were examined, and the key factors influencing the relative mobility of the nitro group, such as the nitrotriazole substrate constitution, the carbon skeleton length of the *O*-nucleophilic agent and the process conditions, were discussed. Several independent competitive reactions directed towards the substitution of the nitro group at position C(5) in the alkyltriazole substrate by different types of nucleophiles such as alkoxide-, hydroxide- and triazolonate anions were observed to take place under conditions used. The major reaction yielded oligoethers containing terminal alkyltriazole heterocycles. Secondary reactions occurred to form the corresponding triazolone and N–C triazolyl triazolone structures in the reaction system. Additionally, in excess of the alkaline agent, alkaline hydrolysis was observed to proceed at the final stages of the process involving the *O*-nucleophile having a longer oligoether backbone in the series studied, leading to the formation of new *O*-nucleophilic sites. The obtained findings can provide a foundation for devising a method for the modification of a wide range of commercially available aliphatic oligo- or polyethers to prepare functional macromolecules whose terminals carry bioactive 1,2,4-triazole heterocycles located at a desired distance from each other.

## 1. Introduction

The interest in aliphatic oligo- or poly-ethers such as oligo- or poly-ethylene glycols (PEGs) has been stimulated in recent times via their successful application in the field of designing new bioactive compounds and modifying various biomolecules [1,2]. PEG is a commercially available, well-water-soluble compound that is chemically inert towards biological structures, and its safety in use is validated by the US Food and Drug Administration (FDA) [3]. The PEG nomenclature is vast and distinguished basically by the carbon skeleton length (the number of elementary monomer units of ethylene oxide) or molecular weight. These merits and the fairly reactive OH groups present on the terminals of the linear macromolecule make PEG an attractive polymer platform for the immobilization of various bioactive compounds [4,5,6,7,8,9,10,11,12,13,14].

**Figure 1:**
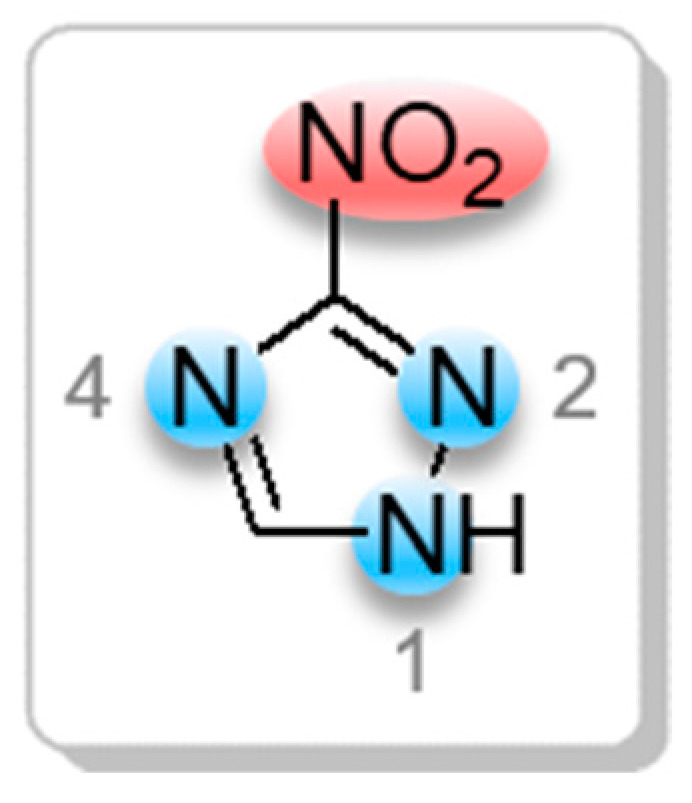
Reactive sites in a 3-nitro-1,2,4-triazole molecule.

Five-membered nitrogen heterocycles such as 1,2,4-triazole derivatives are a unique class of compounds that highly appeal to researchers due to their broad spectrum of pharmacological and biological activities, including antibacterial, antifungal, anti-inflammatory, and others [15,16,17,18,19,20,21,22]. Additionally, 1,2,4-triazole nitro derivatives (for example, 3-nitro-1,2,4-triazole) are quite valuable intermediates for organic synthesis because they can be chemically transformed into other useful compounds being employed to produce practically important, functional materials of many uses [23]. This type of building block offers the advantages of having a great number of reactive sites for the incorporation of different substituents, and these sites can be functionalized through electro- and nucleophilic substitution reactions [24,25,26,27]. For instance, the 3-nitro-1,2,4-triazole molecule has three endocyclic reactive sites—nitrogen atoms (N(1)-, N(2)- and N(4)-)—and one more, quite reactive, exocyclic NO_2_ group (Figure 1).

Such a starting polyfunctional synthon is capable of diverse chemical transformations and opens up broad opportunities for the molecular design of compounds based thereon. The alkylation reaction is a common and quite well-studied method for the functionalization of 1,2,4-triazole nitro derivatives, and all the three nitrogen heteroatoms of the 1,2,4-triazole ring can be the object of an attack by the corresponding alkylating agent, depending on the reaction conditions [24,25,26,27]. The forth exocyclic reactive site on the carbon atom in the 3-nitro-1,2,4-triazole molecule is functionalized via the nucleophilic substitution of the NO_2_ group by various *O*- and *N*-nucleophilic agents [28,29]. However, the *S_N_^ipso^* substitution reaction of the nitro group and the key reaction pathways and the parameters governing the nitro group activity in this type of reaction remain underexplored among N-alkyl-substituted nitro derivatives of 1,2,4-triazole.

This way, from the perspective of basic research, the interest in 1,2,4-triazole derivatives stems from broad opportunities for the molecular and structural design of compounds based on 1,2,4-triazole, which in turn offers prospects for creating tailor-made functional materials. Furthermore, a study on the regularities and behavioral features of polyfunctional 1,2,4-triazoles in underexplored chemical processes is of great interest from both the theoretical and practical perspective.

A literature overview on the modification of the PEG structure towards the formation of triazole moieties in the macromolecule demonstrated that the azide-alkyne cycloaddition is a quite common method for this purpose [30,31,32,33,34,35]. This method generates vicinal 1,2,3-triazole heterocycles in the modified oligoether structure. Since no studies have been found in the available literature sources regarding the incorporation of symmetric 1,2,3-triazole rings into the PEG macromolecule, the present study aimed to investigate the structural modification of aliphatic oligoethers via the *S_N_^ipso^* substitution of the nitro group in 1-alkyl-5-nitro-1,2,4-triazole molecules. Using ^1^H NMR spectroscopy, we examined the relative mobility of the exocyclic NO_2_ group, the key pathways of the process and the product composition from competitive reactions, depending on the temperature–time parameters, ratios of the starting reactants, alkyl substituent types in the nitrotriazole substrate (in the series of methyl-, ethyl- and *iso*-propyl), and carbon skeleton length of the *O*-nucleophilic agent (in the series of mono-, di-, tri- and poly-ethylene glycols).

The findings obtained herein will help to sum up general regularities of the reaction examined, reaction applicability limits, and the forecast of the synthesis feasibility depending on the structural factors of both chemical partners.

## 2. Materials and Methods

The starting 1-methyl-, 1-ethyl- and 1-isopropyl-5-nitro-1,2,4-triazoles were prepared via the alkylation of 3-nitro-1,2,4-triazole with dimethyl and diethyl sulfate, and isopropyl bromide, respectively, in an alkaline medium followed by the isolation of the target compound from mixed isomeric products via the procedures reported in [27]. The other chemicals were procured from Ningbo Mos Chemical Co., Ltd. (Ningbo, Zhejiang, China) and Acros Organics (Morris Plains, NJ, USA), and used as received.

The interaction process between the starting substrates and *O*-nucleophiles was monitored and the composition of the resulting reaction mixture was analyzed via ^1^H NMR spectroscopy. The reaction mass was sampled at specific time intervals, and ^1^H NMR spectra were recorded using a Bruker AV-400 instrument (Bruker Corporation, Billerica, MA, USA) operating at 400 MHz. To confirm the structure of the resulting compounds, we additionally recorded ^13^C NMR spectra at 100 MHz. DMSO-d6 signals (δ 2.5 ppm for ^1^H nuclei and 39.5 ppm for ^13^C nuclei) were used as the internal standard.

### General Procedure for Interaction Process between 1-Alkyl-5-nitro-1,2,4-triazole and Aliphatic Oligoethers (Standard Conditions)

A mixture of 1-alkyl-5-nitro-1,2,4-triazole (with varying alkyl substituents: methyl-, ethyl- and *iso*-propyl) and an aliphatic oligoether (including mono-, di-, tri- and poly-ethylene glycols) in equimolar ratio, dissolved in N,N-dimethylformamide (with a concentration of 1 mol of nitrotriazole substrate per 50 mL), was prepared. A calculated quantity of the alkaline agent (KOH) was then added portion-wise with stirring at 70 °C, given a substrate-to-alkali ratio of 1.0/1.0 mol. KOH was dispensed in several portions (each portion representing 25 mol % of the equimolar ratio) due to the process having an exothermic nature. After every alkali portion added, the reaction mass was sampled in 5 min for subsequent ^1^H NMR analysis. Once alkali dispensing was completed, the reaction mass was additionally stirred at 100 °C for 60 min. Upon the completion of the process under the said temperature–time conditions, the resultant reaction mass was sampled again for ^1^H NMR analysis.

In the case that PEG-1500 was used as the *O*-nucleophilic agent to activate the nucleophilic substitution of NO_2_ groups, the standard conditions described above were modified by varying the temperature and time (100–120 °C; 60–180 min), the ratio of reactants (excess substrate and alkali) and the alkali type (NaOH), as detailed in the Results and Discussion section.

## 3. Results and Discussion

Considering the intensive works on creating bioactive compounds based on polymer macromolecules, we deemed it quite promising to investigate the formation reaction of such compounds involving PEG with varied carbon skeleton lengths as a polymer platform to incorporate symmetric alkyl derivatives of triazole as one of the promising and most in-demand functional bioactive synthons.

The NO_2_ group unsubstituted on the ring heteroatom in 3-nitro-5-R-1,2,4-triazole derivatives is typically inert towards the action of nucleophilic agents. The alkyl substituent being inserted into the nitrotriazole ring considerably favors the activation of the NO_2_ group. The properties and relative stability of isomeric N-alkyl derivatives of C-nitro-1,2,4-triazole in different processes are defined by the structural arrangement of a substrate. The activity of isomeric nitrotriazoles in different types of reactions, including the nucleophilic substitution of the nitro group, differs depending on the position and nature of the alkyl substituent on the heterocyclic nitrogen atom. The alkyl substituent located at the α-position of the nitrotriazole heterocycle (an N(2) isomer) promotes the activation of the exocyclic NO_2_ group to the greatest extent among the N(1)-, N(2)- and N(4)-isomeric derivatives of C-nitro-1,2,4-triazole. On the other hand, the enhancements in electron donor properties and bulkiness of the alkyl substituent of the ring must lead to a reduction in the relative mobility of the nitro group.

Taking into account these considerations, we chose to use the N(2) isomeric alkylnitrotriazoles (Alk-NTZ) as starting substrates because they are the most stable and considerably more active in nucleophilic substitution reactions compared to the other isomeric N-alkyl-C-nitro-1,2,4-triazole derivatives. The starting substrates differed in the type of alkyl moiety substituted on the N(2) atom into the triazole heterocycle in the series of primary 1-methyl-5-nitro-1,2,4-triazole (Me-NTZ) and 1-ethyl-5-nitro-1,2,4-triazole (Et-NTZ), and secondary 1-*iso*-propyl-5-nitro-1,2,4-triazole (*i*-Pr-NTZ).

On the other hand, the choice of a nucleophilic agent also has a significant effect on the nitro group substitution reaction rate and mechanism. The nucleophilicity (activity) of an agent is defined by its ability to generate an active anionic form because a negatively charged nucleophile is always stronger than its conjugated neutral form. To evaluate the relative mobility of the nitro group when *S_N_^ipso^* is substituted by varied *O*-nucleophilic agents, we used a range of glycol derivatives with bifunctional hydroxyls as the *O*-nucleophilic agents: aliphatic oligoethers such as mono (EG), di (DEG) and triethylene glycol (TEG), and polyethylene glycols such as 300 (PEG-300), 400 (PEG-400), 600 (PEG-600) and 1500 (PEG-1500). The chosen oligoethers differed in carbon skeleton length (*n*) or, otherwise speaking, in the number of elementary monomer units of ethylene oxide (EO), and therefore had different abilities to generate active anions under the same conditions in the presence of an alkali. Among the glycols examined, the parameter *n* varied from 1 to 35 when shifting from EG to PEG-1500. The quite-reactive terminal OH groups contained in the oligoethers and located at a different distance from each other according to the parameter *n* make PEGs an attractive polymer platform for embedding various active groups or synthons thereto, depending on the intended use. In our case, combining two alkyltriazole heterocycles into a single oligoether chain would allow us to accumulate positive properties of 1,2,4-triazoles.

The activity of the chosen objects during *S_N_^ipso^*-substitution was evaluated through the reaction performed under equal standard conditions chosen for all the reactants, as detailed in the Materials and Methods section. The interaction between Alk-NTZ and *O*-nucleophilic agents was carried out in aprotic DMF solvent in the presence of alkali the an equimolar ratio of the starting reactants, with the base added portion-wise. The process was monitored from the variation in pH of the reaction mass. The pathway of the nucleophilic substitution of the nitro group on the alkyltriazole heterocycle and the product composition were determined by sampling and analyzing the reaction mass samples using ^1^H NMR spectroscopy both during the portion-wise addition of the alkali and upon completion of the reaction.

**Figure 2:**
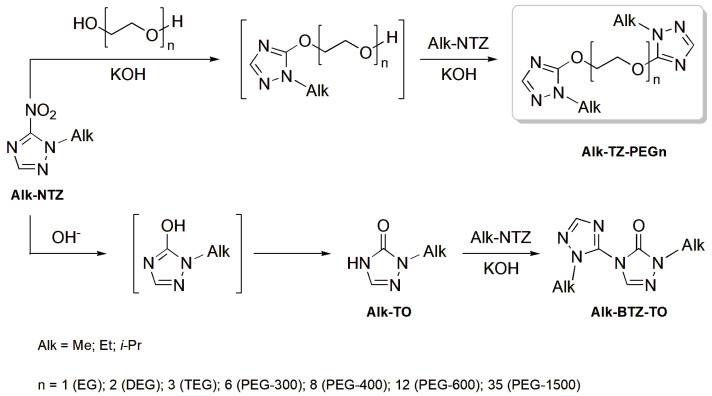
A scheme of competitive reactions of the *S_N_^ipso^* substitution process of the NO_2_ group in Alk-NTZ by aliphatic oligoethers in the presence of an alkali.

Consequently, the interaction between Alk-NTZ and the *O*-nucleophilic agent in the presence of the alkaline agent under the conditions used was found via ^1^H NMR spectroscopy to proceed in several independent directions. The competitive reactions of the process under study are schematically depicted in Figure 2.

First of all, the target reaction of the nucleophilic substitution of the nitro group in the starting substrate by the corresponding O-nucleophilic agent occurred: the C(5) position in the 1-alkyl-1,2,4-triazole molecule was attacked by the oligoether anion (the alkoxide anion formed by the reaction of glycol with the base to detach the NO_2_ group that was detectable as a nitrite anion (KNO_2_ or NaNO_2_) in the reaction medium. The process proceeded through a consecutive parallel formation of products from an incomplete monosubstitution of the oligoether chain (monoalkyltriazole ethers of glycols) and of disubstituted target compounds, in which case the content of monoalkyltriazole ethers of glycols being primarily formed in the reaction system under the conditions used did not exceed 35%. This resulted in a modified oligoether structure with symmetric terminal 1-Alk-1,2,4-triazole moieties of the respective constitution (Alk-TZ-PEGn, where *n* varies from 1 to 35) (Figure 2).

The process of nucleophilic substitution under the adopted standard conditions exhibited its distinct features depending on the type of alkyl substituent in the triazole substrate and on the carbon skeleton length of the oligoether used.

**Figure 3:**
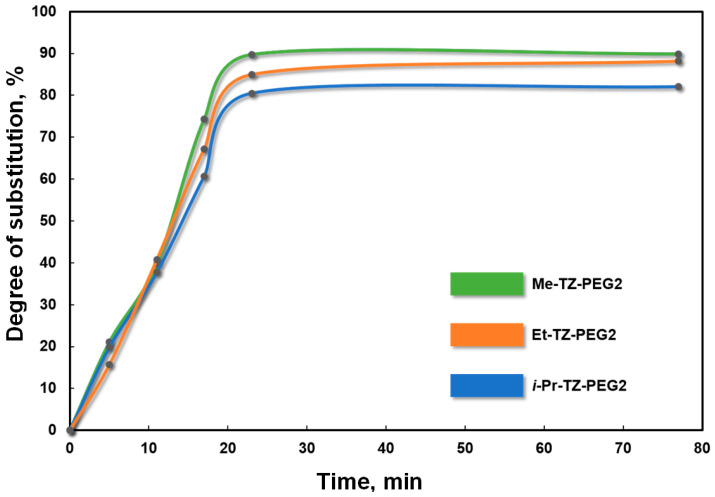
Effect of the type of alkyl substituent in the triazole substrate (in the studied series of Me-NTZ, Et-NTZ and *i*-Pr-NTZ) on the degree of substitution of OH groups in di-ethylene glycol during *S_N_^ipso^* substitution.

The activity of the NO_2_ group was found to depend on the type of alkyl substituent. The enhanced electron donor properties and steric effects of the substituent on the nitrogen atom of the triazole ring lowered the activity of the nitro group towards the same *O*-nucleophilic agent when shifting from the primary type (methyl-; ethyl-) to the secondary one (*iso*-propyl). For instance, the degree of substitution of the terminal OH groups in diethylene glycol by triazole heterocycles when Me-NTZ was used in the presence of KOH in an equimolar ratio of the starting reactants at 100 °C for 77 min was 89.9% (the formation of the disubstituted product Me-TZ-PEG2, Figure 3). Under other equal reaction conditions, the change of the primary methyl substituent in the triazole substrate with the primary ethyl one (the Et-NTZ substrate) and by the secondary *iso*-propyl one (*i*-Pr-NTZ) reduced the degree of substitution to 88.2% (Et-TZ-PEG2) and 82.1% (*i*-Pr-TZ-PEG2), respectively (Figure 3). Thus, under the conditions used (with equal temperature–time process parameters), the activity of the NO_2_ group in the nucleophilic substitution reaction involving oligoethers and the alkaline agent diminished in the order of Me-NTZ > Et-NTZ > *i*-Pr-NTZ, depending on the type of alkyl substituent on the nitrotriazole ring.

**Figure 4:**
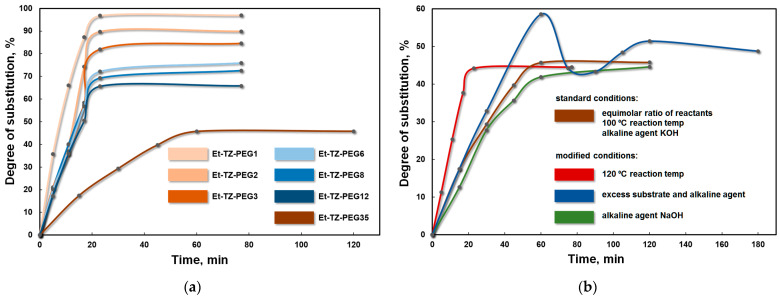
The degree of substitution of OH groups in the oligoether macromolecule by ethylnitrotriazole heterocycles plotted against (**a**) the carbon skeleton length of the *O*-nucleophile in the studied series under standard conditions and (**b**) under modified conditions involving PEG-1500.

The carbon skeleton length (*n*) or the number of elementary monomer units of ethylene oxide (EO) in the aliphatic oligoether had a significant impact on the activity of the nitro group in the triazole substrate. The reactions involving 1-ethyl-5-nitro-1,2,4-triazole as the starting substrate demonstrated that the degree of conversion of terminal groups of the macromolecule into alkyltriazole heterocycles was observed to decline as the oligoether chain of the *O*-nucleophilic agent increased in length (Figure 4a). The process under study proceeded most actively when EG (*n* = 1), the first representative of the examined series of oligoethers, was used, and the degree of substitution (otherwise speaking, the formation of disubstituted product Et-TZ-PEG1) reached 97.0%. Under similar experimental conditions, the change of monoethylene glycol with DEG (*n* = 2) slightly decreased the relative mobility of the NO_2_ group during the reaction, thereby affecting the degree of substitution which declined to 89.9% (Et-TZ-PEG2). The degree of substitution of terminal OH groups diminished to 84.6% (Et-TZ-PEG3) when the macromolecule consisting of three EO monomer units (TEG, *n* = 3) was used. In the case that the oligomeric (at *n* > 3) structural analogues were employed as the *O*-nucleophiles, the mobility of the nitro group during the substitution was decreased more significantly compared to that under the use of low-molecular (at *n* ≤ 3) glycols described above. For instance, when oligoethers with the carbon skeleton length of 6 (PEG-300) and 8 (PEG-400) EO units were put into the reaction, the degrees of substitution were 75.9% (Et-TZ-PEG6) and 72.6% (Et-TZ-PEG8), respectively (Figure 4a).

A further increase in the number of ethylene oxide units in the macromolecular structure to 12 (PEG-600) lowered the degree of substitution to 65.8% (Et-TZ-PEG12). The similar standard conditions involving the *O*-nucleophile with the longest carbon skeleton in the studied series (*n* = 35, PEG-1500) failed to attain even a 50% degree of substitution (45.9%, Et-TZ-PEG35).

The specially performed experiments with PEG-1500 under the modified conditions (Figure 4b) in order to increase the degree of substitution of NO_2_ groups by varying the type of alkaline agent (KOH changed to NaOH), process temperature (increased from 100 °C to 120 °C) and time (between 77 and 180 min) did not considerably alter the degree of substitution whose value almost remained at the same level of 44.5% (Figure 4b). On one hand, the stepwise addition to the reaction medium of a twofold excess of the starting Et-NTZ and KOH with respect to PEG-1500 allowed the conversion degree to be raised to 58.5%. On the other hand, the excess alkaline agent present in the reaction system seemingly developed destructive processes of the oligoether backbone of the target products secondary to the alkaline hydrolysis reaction, leading to the formation of new *O*-nucleophilic sites. As seen in Figure 4b, excess Et-NTZ and KOH initially promoted the activation of NO_2_ groups in the substrate, i.e., the curve showed an increase in the degree of substitution over time. However, an abrupt decline was observed after 58.5% was attained and might evidence the breaking of the ether bond of the backbone of the compounds resulting from the target *S_N_^ipso^* substitution reaction of the nitro group.

On one hand, the established relationship between the *S_N_^ipso^* substitution of the nitro group in the alkylnitrotriazoles and the carbon skeleton length of the *O*-nucleophile (Figure 4a) may be indicative of some inner regularities typical of the oligoether chain. On the other hand, it may indicate structural changes occurring directly within the modified macromolecule.

The terminal OH groups of the initial aliphatic oligoethers are capable of forming intra- and intermolecular hydrogen bonds to the ether oxygen atoms in the backbone. Depending on the carbon skeleton length or the number of ethylene oxide monomer units, the oligoether structure will undergo changes in the number of these associated moieties and, in one way or another, hydrogen bonding. In our case, when shifting from the first representatives of the examined class of *O*-nucleophilic agents (at *n* ≤ 3: EG, DEG and TEG) to the long-chain structural analogues (*n* > 3: PEG-300, PEG-400, PEG-600 and PEG-1500), the associated moieties considerably increased in number due to an increase in the repeat ether moieties (EO) of the backbone from 1–3 to 6–35. It is reasonable to assume that the free hydroxyl groups were the most active and were the first to participate in the *S_N_^ipso^* substitution reaction of the nitro group, while as the reaction was progressing, the equilibrium shifted towards unassociated OH groups and the process slowed down appreciably. With the increasing carbon skeleton length of the oligoether, the hydroxyls generating hydrogen bonds became sterically less available for the nitrotriazole substrate. The aforesaid is mirrored in the graphical relationship shown in Figure 4a. It is seen that the process proceeded most actively with EG but slowed down noticeably when switching to PEG-1500. Overall, as the process was progressing, nucleophilic substitution was initially observed to develop fast under the conditions adopted, irrespective of the *O*-nucleophile used, and gradually came out to quite a stable plateau at the final reaction stages (Figure 4a). That said, the initial substrate was almost completely consumed (except for the experiments with PEG-1500) within 30–60 min at 100 °C, and the content of monoethyltriazole glycol ethers (monosubstitution products) being primarily formed in the reaction system was small, with a considerable content of the target product Et-TZ-PEGn, suggesting the higher nucleophilic activity of the salt forms of monoethyltriazole ethers of the glycols compared to that of the anions of the original unmodified glycols. The revealed feature is in agreement with the study results on the substitution of OH groups of glycols (in the series of mono-, di- and tri-ethylene glycol) when a nitroaromatic substrate was used [36].

The above-described regularities refer to the major reaction from among the detected independent competitive reactions of the process examined. Regardless of the process conditions and characteristics of the starting reactants, all the experimental samples from the reaction mixtures were found via ^1^H NMR spectroscopy to contain triazolone derivatives of varied structural arrangements. The formation of these derivatives indicates the progress of secondary reactions. The use of the alkaline agent as a catalyst for the *S_N_^ipso^* substitution of the NO_2_ group in the substrate molecule resulted in an additional insertion of the hydroxide anion into the reaction system. Consecutively and in parallel to the major reaction between the substrate and the *O*-nucleophilic agent, there occurred an interaction between the starting 1-alkyl-5-nitro-1,2,4-triazole and hydroxide anion present in the system. As a result, 1-alkyl-1,2,4-triazol-5-one (Alk-TO) was formed in the reaction system (Figure 2). Unlike the major reaction of the nucleophilic substitution of the nitro group by the alkoxide, the secondary reaction of the substitution of the nitro group in the substrate by the alkoxide anion is a typical tandem transformation. Due to the presence of a quite highly reactive endocyclic site (NH group) in its structure, the resulting Alk-TO can itself function as an N-nucleophilic agent and participate in a heterolysis reaction, attacking the C(5) position of the starting Alk-NTZ substrates in the form of a triazolonate anion. The source for triazolone anions was the alkaline agent which was consumed for the generation of both alkoxide, hydroxide and basically triazolonate anions under the process conditions. As a result of the interaction between Alk-NTZ and Alk-TO in an activated anionic form, the reaction system was observed to consume triazolone and generate bicyclic triazolyl triazolone derivatives with an N–C bond between the heterocycles, regardless of the oligoether used in the major reaction (Figure 2): 2,2′-dialkyl-2H,2′H-[3,4′]bi([1,2,4]triazolyl)-3′-one (Alk-BTZ-TO). Reactions of this kind forming bicyclic structures are typical of most of the nitro derivatives of 1,2,4-triazole substrates when they interact with various 3-R-5-R1-1,2,4-triazolides acting as N-nucleophilic agents in this case [28].

**Figure 5:**
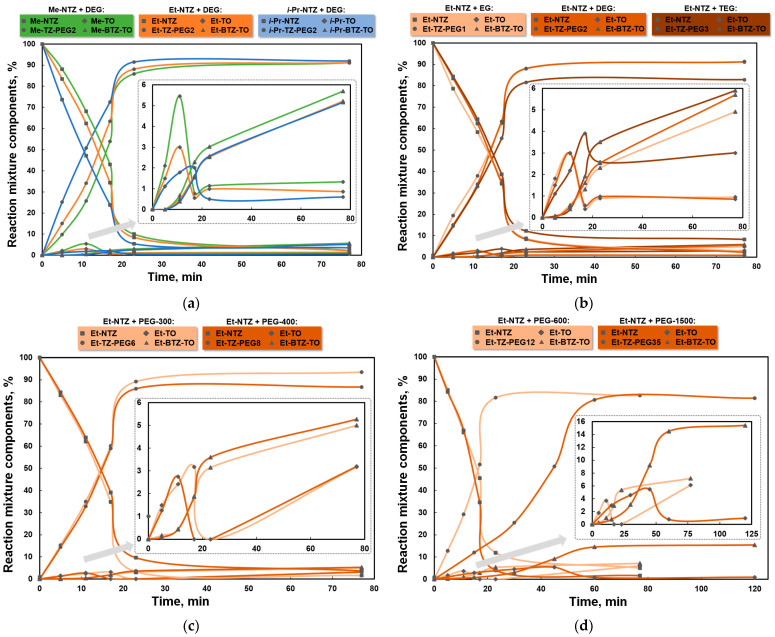
The composition of reaction mixtures subject to (**a**) the alkyl substituent in the substrate involving di-ethylene glycol, and the carbon skeleton length of the *O*-nucleophilic agent involving Et-NTZ and oligo-ethers (**b**) in the series of EG–TEG, (**c**) in the series of PEG-300–PEG-400 and (**d**) in the series of PEG-600–PEG-1500.

Overall, the accumulation of the target and secondary products over time suggests that the nucleophilic substitution reaction of the NO_2_ group using alkoxide anions (the target substitution) prevailed over secondary and parallel reactions involving hydroxide- and triazolonate anions (Figure 5a–d). That is, the alkaline agent was primarily consumed most notably for the formation of an alkoxide anion in the reaction with the corresponding glycol. By interacting with the starting substrate, the salt form of glycol was further activated via the attachment of the alkyltriazole molecule, leading to an acceleration of the subsequent attachment of the substrate to the polyether chain. That said, the reaction system was observed to decrease in the proportion of Alk-NTZ and increase in the proportion of the major reaction product Alk-TZ-PEGn (Figure 5a–d).

In parallel to the main process, the interaction between the hydroxide anion and the substrate resulted in the formation and accumulation of a secondary product of alkyltriazolone as an N-H form, whose cyclic proton on the carbon atom can be clearly detected in the NMR spectra in a separate region devoid of overlapped proton signals from other compounds. The revealed relationship between the nitro group’s activity and the type of alkyl substituent in the main interaction process with alkoxide anions was also seen in the secondary competitive reactions. For instance, depending on the type of alkyl moiety substituted over the endocyclic N(2) atom in the substrate, it can be seen from the graphical dependence of an extreme nature in Figure 5a that the maximum proportion of the secondary reaction product Alk-TO in the reaction mixture involving DEG as the *O*-nucleophile can reach 5.5% at the early stages of the process within the specified timeframe in the case that the most active substrate Me-NTZ is involved in the nucleophilic substitution reaction. The replacement of the primary methyl substituent by the primary ethyl and secondary substituents reduced the proportion of Alk-TO to 3.0% and 2.0%, respectively.

As the major *S_N_^ipso^* substitution reaction of the NO_2_ group in the substrate molecule by the corresponding glycol began to slow down—possibly due to changes in the acidic/basic properties of the newly formed products and in the ratio of equilibrium concentrations between the compounds found in the reaction system—the proportion of Alk-TO was decreased by its interaction with the free alkaline agent and by the transformation of the N-H form into the salt one. In turn, when interacting with each other, the activated triazolonate anion and the starting substrate generated other secondary products of a bicyclic arrangement. The proportion of the said bicyclic derivatives, Alk-BTZ-TO, resulted from the ionized alkyltriazolone being gradually consumed for its interaction with the starting Alk-NTZ was accumulating throughout the entire reaction and could reach 5.7% (for Me-NTZ), 5.2% (for Et-NTZ) and 5.1% (for *i*-Pr-NTZ) under the conditions used, depending on the substrate’s activity.

A similar tendency was observed when Et-NTZ reacted with the corresponding *O*-nucleophilic agent in the series studied (Figure 5b–d). Along with that, under process conditions with low-activity oligoether terminal groups (when EG was changed to PEG-600 and especially to PEG-1500), the proportion of products from secondary competitive reactions reasonably increased in the reaction mixture because the target substitution reaction slowed down. For instance, as the reaction time was being extended, the maximum proportion of Et-TO was 3.0–3.9% (for the EG–TEG series, Figure 5b), 3.1–3.2% (for the PEG-300–PEG-400 series, Figure 5c) and 3.7–5.5% (for the PEG-600–PEG-1500 series, Figure 5d). The proportion of the bicyclic compounds, Et-BTZ-TO, in the reaction mixture increased throughout the entire process and reached 4.9–5.9% (for the EG–TEG series), 5.0–5.3% (for the series PEG-300–PEG-400) and 7.2–15.4% (for the series PEG-600–PEG-1500), depending on the *O*-nucleophilic activity.

It should be noted that under the process conditions examined, both the starting Alk-NTZ substrates and the resultant target Alk-TZ-PEGn and secondary Alk-TO and Alk-BTZ-TO compounds did not undergo heterolysis via the bond that links the triazole ring to the functionalized N-alkyl substituent. The type of alkyl group for all the compounds remained similar to that of the starting substrate involved in the process.

As mentioned earlier herein, ^1^H NMR spectroscopy was used to confirm the structure of the resulting compounds and determine the product composition from the above-described competitive reactions of the examined process of *S_N_^ipso^*-substitution of the NO_2_ group by aliphatic oligoethers.

The signals from the ring protons on the C(3) atom for the starting substrates (Alk-NTZ) and major reaction (Alk-TZ-PEGn) and competitive reaction (Alk-TO, Alk-BTZ-TO) products differed greatly in chemical shifts in their spectra, making it possible to control the processes of consumption of starting reactants, accumulation of target products, transformation of intermediates and, on the whole, evaluate the changes in the product composition from competitive reactions over time throughout the entire process.

**Figure 6:**
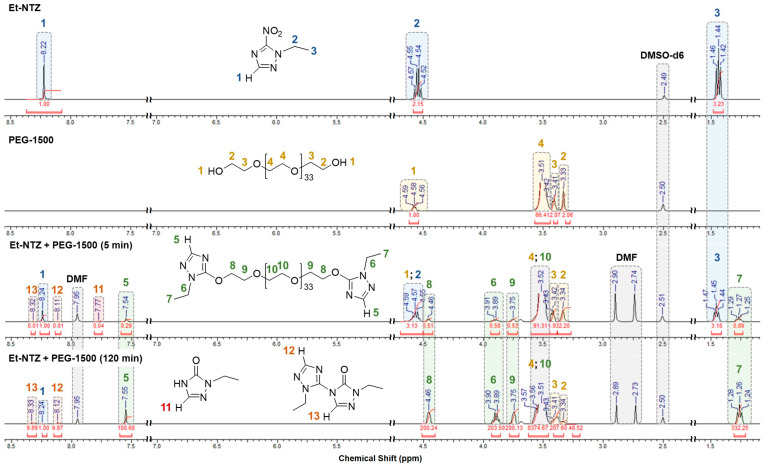
^1^H NMR spectra of the starting reactants Et-NTZ and PEG-1500 and of reaction mixtures sampled 15 min and 120 min after their interaction.

Figure 6 illustrates typical proton NMR spectra of the starting reactants and reaction mixtures sampled at the onset (15 min) and endset (120 min) of the process, as exemplified by the interaction between Et-NTZ and PEG-1500.

Compared to the proton signals from the starting substrate, those from the terminal ethyltriazole groups of the target product Et-TZ-PEG35 from the interaction of Et-NTZ with PEG-1500 are shifted upfield due to the triazole ring missing an electron-withdrawing NO_2_ group (due to its substitution). For instance, the ring protons on the C(3) atoms of the terminal heterocycles (5 in Figure 6) are detected as a single singlet at 7.54–7.55 ppm (instead of 8.22–8.24 ppm (1) for Et-NTZ), and the proton signals from the ethyl substituent substituted over the heterocyclic nitrogen atom show up as a qaudruplet (–CH_2_–CH_3_, 6) and a triplet (–CH_2_–CH_3_, 7) at 3.89–3.90 ppm (instead of 4.54–4.55 ppm (2)) and 1.26–1.27 ppm (instead of 1.44–1.45 ppm (3)), respectively (Figure 6). The proton signals from the major repeat structural elements (EO monomer units) of the oligoether chain in the modified Et-TZ-PEG35 macromolecule (8–10) are shifted upfield as they move away from the heterocycle, depending on their location with respect to the terminal alkyltriazole rings. The protons of the CH_2_–CH_2_ groups directly linked to the terminal triazole rings via the ether oxygen show up at 4.46 ppm (Et-TZ–O–CH_2_–CH_2_, 8) and 3.75 ppm (Et-TZ–O–CH_2_–CH_2_, 9). The protons of the central elements of the oligoether chain are detected as a broad signal at 3.51–3.52 ppm (10) and overlap with the analogous proton signals from PEG-1500 (4). The proton signals from the CH_2_–CH_2_ groups linked to the terminal OH groups of the starting *O*-nucleophilic agent are observed upfield at 3.33–3.34 ppm (HO–CH_2_–CH_2_, 2) and 3.41–3.42 ppm (HO–CH_2_–CH_2_, 3), and the intensity of these signals decline as the latter are being substituted (Figure 6).

For the secondary products, the chemical shift of the C(3)–H proton in the Et-TO triazolone is observed as more upfield at 7.77 ppm (11) relative to the ring proton signal from the starting substrate and as more downfield relative to signals from the target structure. As the process was progressing, the Et-TO was activated to transition to an ionized form and consumed for the generation of a bicyclic derivative. This is supported by the presence and gradual increase in two equally intense characteristic signals of the cyclic protons in the downfield region at 8.11–8.12 ppm (12) and 8.32–8.33 ppm (13) in the spectra of the reaction mixture, evidencing the formation of the N–C bicyclic compound, Et-BTZ-TO.

**Figure 7:**
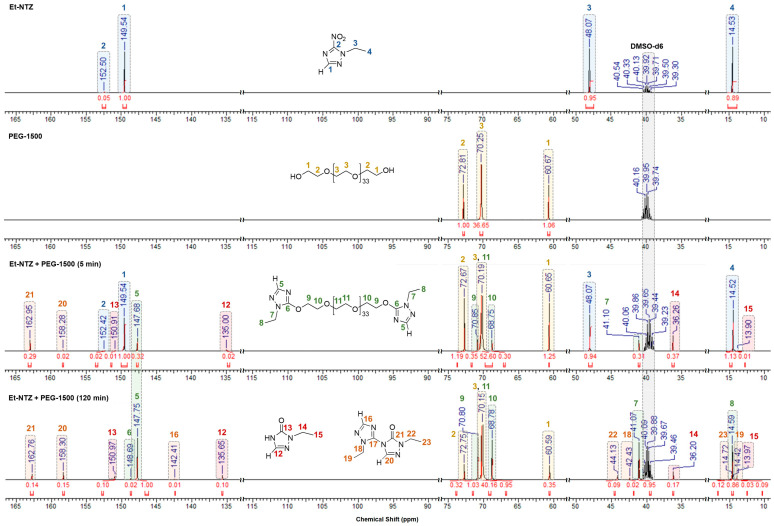
^13^C NMR spectra of the starting reactants and of reaction mixtures sampled 15 min and 120 min after their interaction.

The structures of the compounds resulting from the process in question were additionally confirmed via ^13^C NMR spectroscopy (Figure 7). Because of the substitution of the NO_2_ group in the Et-NTZ molecule, the signals from the endocyclic C(3) and C(5) atoms of the terminal 1,2,4-triazoles, as well as from the ethyl substituent for the major reaction product, are shifted more upfield in the ^13^C spectra relative to the analogous signals from the starting substrate. For instance, the heterocyclic C(3) atoms for Et-TZ-PEG35 are observed at 147.68–147.75 ppm (5 in Figure 7), whereas the analogous carbon signal for the starting Et-NTZ is at 149.54 ppm (1 for Et-NTZ). The C(5) signal associated with the nitro group is poorly visible in the spectrum near 152.50 ppm (2 for Et-NTZ) for the substrate because of the quadrupolar broadening observed typical of azole nitro derivatives [37]. After the NO_2_ group was substituted, the C(5) atoms of the terminal triazole rings related to the oligoether matrix are recorded at 148.69 ppm (6 in Figure 7). The resonance of the carbon atoms of the ethyl substituent associated with the triazole ring are located at 41.07–41.10 ppm (–CH_2_–CH_3_, 7) and 14.59 ppm (–CH_2_–CH_3_, 8) for Et-TZ-PEG35, instead of 48.07 ppm (–CH_2_–CH_3_ for Et-NTZ, 3) and 14.52–14.53 ppm (–CH_2_–CH_3_ for Et-NTZ, 4). The carbon atoms of the oligoether chain of the macromolecule of compound Et-TZ-PEG35 are detected as three characteristic resonance signals at 70.80–70.85 ppm (Et-TZ–O–CH_2_–CH_2_, 9), 68.75–68.78 ppm (Et-TZ–O–CH_2_–CH_2_, 10) and the most intense one at 70.15 ppm (related to the central carbon atoms of the repeat units of O–CH_2_–CH_2_, 11). The intensity of the said signals for Et-TZ-PEG35 rises as the transformation of the NO_2_ groups increases when the Et-NTZ substrate interacts with *O*-nucleophilic agent PEG-1500.

The signals from the endocyclic carbon atoms related to the secondary triazolone and bicyclic products from the process under study are detected in the characteristic downfield region of the ^13^C spectrum. For Et-TO, the C(3) atom is located at 135.00–135.65 ppm (12 in Figure 7) in the ^13^C spectrum, and the carbon of the C=O carbonyl is noticed at 150.91–150.97 ppm (13 in Figure 7). For bicyclic Et-BTZ-TO, the carbon atoms of the ethyltriazole moiety are detected at 142.41 ppm (C(3)–H, 16) and 150.61 ppm (C–N, 17 in Figure 7). For the ethyltriazolone moiety of the Et-BTZ-TO molecule, the signals from the endocyclic carbon atoms are observed more downfield at 158.28–158.30 ppm (C(3)–H, 20) and 162.76–162.95 ppm (C=O, 21).

The carbon atoms of the alkyl substituents for the secondary reaction products are recorded near 36.20–44.13 ppm (–CH_2_–CH_3_, 14 for Et-TO and 18, 22 for Et-BTZ-TO) and 13.90–14.72 ppm (–CH_2_–CH_3_, 15 for Et-TO and 19, 23 for Et-BTZ-TO) in the spectrum.

## 4. Conclusions

As a result of the ^1^H NMR spectroscopic study performed, we have investigated the main pathways of the *S_N_^ipso^* substitution process of the nitro group in 1-alkyl-5-nitro-1,2,4-triazole molecules using aliphatic oligoethers in the presence of an alkali, as well as the composition of the resulting products from competitive reactions, and discussed the major factors influencing the relative mobility of the NO_2_ group. Several independent competitive reactions directed towards the substitution of the nitro group at position C(5) in the alkyltriazole substrate by different types of nucleophiles such as alkoxide-, hydroxide- and triazolonate anions were observed to occur under the conditions used. The major reaction yielded the target oligoethers with symmetric terminal alkyltriazole heterocycles. The secondary reactions resulted in the formation of the corresponding triazolone and N-C triazolyl triazolone structures in the reaction system.

## Data Availability

Data are contained within the article.
